# Biophysical studies suggest a new structural arrangement of crotoxin and provide insights into its toxic mechanism

**DOI:** 10.1038/srep43885

**Published:** 2017-03-03

**Authors:** Carlos A. H. Fernandes, Wallance M. Pazin, Thiago R. Dreyer, Renata N. Bicev, Walter L. G. Cavalcante, Consuelo L. Fortes-Dias, Amando S. Ito, Cristiano L. P. Oliveira, Roberto Morato Fernandez, Marcos R. M. Fontes

**Affiliations:** 1 Departamento de Física e Biofísica, Instituto de Biociências, Universidade Estadual Paulista, UNESP, Botucatu-SP, Brazil; 2 Departamento de Física, Faculdade de Filosofia Ciências e Letras de Ribeirão Preto, USP, Ribeirão Preto-SP, Brazil; 3 Departamento de Física Experimental, Instituto de Física, Universidade de São Paulo – USP, São Paulo, SP, Brazil; 4 Departamento de Farmacologia, Instituto de Ciências Biológicas, UFMG, Belo Horizonte, MG, Brazil; 5 Diretoria de Pesquisa e Desenvolvimento, Fundação Ezequiel Dias (FUNED), Belo Horizonte, MG, Brazil

## Abstract

Crotoxin (CTX) is the main neurotoxin found in *Crotalus durissus* rattlesnake venoms being composed by a nontoxic and non-enzymatic component (CA) and a toxic phospholipase A_2_ (CB). Previous crystallographic structures of CTX and CB provided relevant insights: (i) CTX structure showed a 1:1 molecular ratio between CA and CB, presenting three tryptophan residues in the CA/CB interface and one exposed to solvent; (ii) CB structure displayed a tetrameric conformation. This study aims to provide further information on the CTX mechanism of action by several biophysical methods. Our data show that isolated CB can in fact form tetramers in solution; however, these tetramers can be dissociated by CA titration. Furthermore, CTX exhibits a strong reduction in fluorescence intensity and lifetime compared with isolated CA and CB, suggesting that all tryptophan residues in CTX may be hidden by the CA/CB interface. By companying spectroscopy fluorescence and SAXS data, we obtained a new structural model for the CTX heterodimer in which all tryptophans are located in the interface, and the N-terminal region of CB is largely exposed to the solvent. Based on this model, we propose a toxic mechanism of action for CTX, involving the interaction of N-terminal region of CB with the target before CA dissociation.

Crotoxin (CTX) is a β-neurotoxin that is the main protein component in the venoms of South American *Crotalus durissus terrificus* (Viperidae) rattlesnakes. This toxin exerts lethal action through a potent blockade of neuromuscular transmission, mostly at the presynaptic level, preventing acetylcholine release from peripheral neurons at the neuromuscular junction^1^,^2^,^3^.

Native CTX is a heterodimeric complex consisting of a noncovalent association between an acidic non-enzymatic protein (crotoxin A, CA or crotapotin) and a basic toxic phospholipase A_2_ (crotoxin B or CB)^4^,^5^. CA does not display any toxicity itself, but it enhances the pharmacological activity of CB by preventing its adsorption to non-saturable binding sites, thereby restricting its binding to critical target sites at neuromuscular junctions^4^,^6^,^7^,^8^,^9^. However, neither the exact mechanism of action nor the amino acid residues involved in the neurotoxicity of CTX, or of other presynaptic toxins, have been fully understood^10^. In the past, it was believed that CTX acted on plasmatic membranes, initiating events that would culminate in neuromuscular blockade, without internalization^11^. More recent data have shown that presynaptic toxins from snake venoms, in general, enter into the lumen of synaptic vesicles following endocytosis and hydrolyze phospholipids of the inner leaflet of the membrane^12^.

Although neuromuscular blockade by presynaptic activity has been the most studied effect of CTX, postsynaptic activity has been also observed^13^,^14^. Furthermore, CTX shows additional effects, including cardiotoxicity^15^, nephrotoxicity^16^ and myotoxicity^17^,^18^,^19^, being the major contributor to the systemic myotoxicity observed in victims of *C. d. terrificus* bites^20^,^21^. Recently, biological activities with potential therapeutic applications such as immunomodulatory, anti-inflammatory, analgesic and anti-tumor activities have been also described for CTX (reviewed in ref. 10).

In general, it is accepted that both Ca^2+^-dependent phospholipid hydrolysis^22^,^23^ and binding to membrane acceptors may participate in the presynaptic neurotoxicity induced by CTX^24^,^25^,^26^. However, some studies have shown that the catalytic activity alone does not explain the neurotoxicity of CTX, indicating that other regions of CB must be involved in neurotoxicity, such as Tyr22^27^ and the C-terminal region^28^. Indeed, the C-terminus has been indicated to be responsible for the neurotoxicity in ammodytoxin, a β-neurotoxin from *Vipera ammodytes ammodytes* (Viperidae) venom^29^,^30^.

The random association of different isoforms of both CA and CB may result in at least sixteen distinct CTX complexes, which can eventually coexist in a single specimen of *C.d. terrificus* snake^31^. Different isoforms of CB exhibit slight modifications in the enzymatic and pharmacological properties of the CTX, grouping the CTX isoforms into classes I and II^32^. When complexed with CA, the CB_b_, CB_c_ and CB_d_ isoforms are more toxic, have less enzymatic activity and dissociate from CA (class I isoforms) more slowly than CB_a2_ isoform (class II isoforms)^32^.

The crystal structure of isolated CB was obtained using samples containing a natural pool of CB isoforms and showed a tetramer quarternary structure consisting of two dimers, each dimer constituted by a CBc and a CBa_2_ isoform (class I and class II isoforms, respectively)^33^. More recently, the crystal structure of CTX formed by a heterodimeric association between CA and CB molecules was reported using a single isoform of crotoxin (CA_2_CB_b_; class I isoform)^5^. This study provided the first high-resolution model of CA showing that this protein is formed by three disulfide-bonded polypeptide chains (α, β, and γ). The α and β chains are alpha-helices with loops at the terminal positions, and the γ chain is a disordered loop^5^. Additionally, the authors demonstrated that Trp36 from CA, Trp31 and Trp70 from CB are part of the CA/CB interface and play important roles in the stability of the CA/CB complex.

For insight into the behavior of tryptophans in the CA, CB and CTX proteins, we performed static and time-resolved spectroscopy fluorescence as well as time-resolved anisotropy fluorescence experiments. Furthermore, we used cross-linking assays, dynamic light scattering (DLS) and small angle X-ray scattering (SAXS) analyses to understand the quaternary structure of CTX and its isolated subunits in solution. The results provided here show that a new structural arrangement for the CTX heterodimer was obtained, in which the role of tryptophans on CA/CB interface is studied. Furthermore, based on this model and the biophysical experiments performed here, we suggest a toxic mechanism of action involving the N-terminal region of CB.

## Materials and Methods

### Purification of Crotoxin (CTX), Crotoxin A (CA), Crotoxin B (CB) from *Crotalus durissus terrificus*

Crotoxin (CTX) and its subunits (CA and CB) were purified from *Crotalus durissus terrificus* venom by molecular exclusion followed by reversed phase (RP) high-performance liquid chromatography (HPLC) (ÄKTA Purifier 10 system, GE Healthcare), similarly to the methods described in previous studies^34^,^35^. Crude venom (15 mg) was dissolved in 500 μL of ammonium bicarbonate buffer (0.05 M, pH 8.0), then centrifuged at 10,000 rpm for 10 minutes at 4 °C. The supernatant was applied to a Superdex 75 10/300 GL column (GE Healthcare) pre-equilibrated with ammonium bicarbonate buffer (1 M, pH 8.0). The elution was performed at a flow rate of 0.5 mL/min. Fractions 0.5 ml in volume were collected and monitored at 280 nm. CTX fractions were lyophilized and stored at −20 °C. The CA and CB subunits were isolated through RP-HPLC. Two milligrams of CTX were dissolved in 0.1% trifluoroacetic acid (TFA; solvent A) and applied to a C18 column (Sephasil Peptide 4.65 m/250, Pharmacia Biotech) pre-equilibrated with solvent A. The proteins were eluted with a 0–100% linear gradient of 0.1% TFA in 66.6% acetonitrile (solvent B) at a flow rate of 1 mL/min and monitored at 280 nm. The CA and CB fractions were lyophilized and stored at −20 °C until use. All experiments were performed with the natural mixture of CA and CB isoforms present in the purified CTX. For the PLA_2_ activity assay, CA and CB were dissociated by modified RP chromatography as previously described^36^, using a C18 Small Pore 5 μm, 4.6 × 250 mm column (Vydak) instead of a C4. Before to perform the biological and biophysical assays described in the present study, all lyophilized samples (CTX, CA and CB) were dissolved in a buffer (Tris HCl or ammonium formate) and subsequently, a 48 hours dialysis was executed against the same buffer to ensure the removal of TFA and acetonitrile of the solution.

### Circular dichroism spectroscopy

The secondary structural integrity of the purified samples was verified by circular dichroism spectroscopy over the spectral range of 200–260 nm using a JASCO J-815 spectropolarimeter (JASCO Spectroscopic Co., Ltd., Japan) equipped with a Peltier thermo-controller. The experiments were performed at 293 K using an optical path length of 0.5 nm, a scanning speed of 100 nm/min, a response time of 1 s, a bandwidth of 2 nm and a data pitch of 0.5 nm. Twenty spectra of CTX and isolated CA and CB samples were acquired, averaged and corrected for the buffer solution (baseline). The proteins were analyzed at 0.25 mg.ml^−1^ in buffer containing 20 mM Tris HCl pH 8.0.

### PLA_2_ activity assay

The enzymatic activity of CTX before and after fractionation by RP-chromatography was verified by comparing the corresponding PLA_2_ activity curves of the native and reconstituted (by pooling fractions of CA and CB) complex by varying concentrations from 12.5 to 900 μg/ml. The activity was assayed by the egg yolk clearing method^37^, using duplicates for each concentration. PBS was used as a negative control. The data were analyzed by linear regression by the least squares method, using Graph Prism 6.0 for Mac OS X (GraphPad software Inc., La Jolla, California) with 95% confidence.

### Composition of CA and CB in *Crotalus durissus terrificus* venom and in CTX complex

CTX was purified from three different lots of crude *C. d. terrificus* venom: (i) a white variety collect in Queluzito (20°44′7″S, 43°52′36″W); (ii) a yellow variety collect in Carrancas (21°29′15″S, 44°38′33″W) and iii) a lot of the reference venom (Fundação Ezequiel Dias – FUNED, Belo Horizonte, MG, Brazil) used in the preparation of anti-crotalic serum. Two milligrams of each venom was separately load on a Superdex 200 10/300 GL column (GE Healthcare) in an Akta Purifier 10 (GE Healthcare), eluted with 50 mM bicarbonate buffer pH 7.9, at a flow rate of 0.5 ml/min. The CTX-containing fractions were subsequently applied on a C18 RP column, as described in section 2.1. The crude venoms were applied on the same column. The molar ratio between CA and CB in the whole venom and in the corresponding CTX fractions was calculated by integration of the area under the curve of 280 nm absorbance. Molar extinction coefficients at 280 nm based on the average amino acid compositions of the CA and CB isoforms (12,761 cm^−1^M^−1^ and 32,190 cm^−1^M^−1^, respectively) were used in the calculations.

### Cross-linking assays

Ten microliters of glutaraldehyde (25% EM grade, Merck) at increasing concentration was added to 10 μl of CTX (0.3 mM) or CB (0.4 mM) in 20 mM Tris pH 8.0. The mixture was incubated in the dark for 3 h at room temperature before SDS-PAGE analysis on 8–25% gradient Phast^®^ gels (GE Healthcare) under non-reducing conditions. A control reaction without glutaraldehyde was run in parallel.

### Dynamic light scattering

Dynamic light scattering (DLS) measurements were performed at 291 K using the instrument DynaPro TITAN (Wyatt Technology). CTX, CA, CB and reconstituted CTX (obtained by a mixture of CA and CB in a 1:1 molecular ratio) were dissolved in 20 mM Tris HCl pH 8.0 at concentrations of 3 mg.ml^−1^. Data were measured one hundred times, and the results were analyzed using the Dynamics v.6.10 software. The hydrodynamic radius (R_h_) was obtained through the Stokes-Einstein relation *D*_*t*_ = *kT/6πηR*_*h*_, where *D*_*t*_ (translational diffusion constant) is obtained by an autocorrelation function from the data measured, *k* is the Boltzmann constant, *T* is the temperature (Kelvin), and *η* is the solvent viscosity. The estimated molecular mass obtained is based on an empirical curve of known globular proteins and their measured hydrodynamic radius. The polydispersity of the samples is the standard deviation of the particle size distributions from the mean value, weighted by their mass fraction. The percentage of polydispersity (% Pd) is calculated by dividing the polydispersity value by the mean R_H_ multiplied by 100.

### Isothermal titration calorimetry (ITC)

Calorimetric experiments were performed using a microcalorimeter iTC_200_ (GE Healthcare) at 25 °C. Proteins were prepared in 50 mM ammonium formate pH 6.6. CA (240 μM) was titrated (20 injections of 2 μL at each 180 seconds) into the calorimetric cell containing CB (15 μM). Titrations were performed in duplicate, and the heats of mixing and dilution were determined in control experiments and subtracted from the titrations. Data analyses were performed using binding polynomials considering two binding events^38^.

### Steady-state and time-resolved fluorescence spectroscopy and fluorescence lifetime measurements

Static fluorescence spectroscopy measurements of CTX, CA, and CB were recorded using a Hitachi F-7000 spectrofluorimeter with a 1 cm path length cuvette and a bandwidth of 2 nm. The fluorescence emission spectra of tryptophan residues were measured from 215 to 465 nm and from 330 to 350 nm, for static and anisotropy fluorescence measurements, respectively, with an excitation wavelength of 290 nm. Fluorescence intensity decay and time-resolved anisotropy measurements were recorded based on time-correlated single-photon counting techniques. The excitation source was a mode-locked Ti:sa laser (Tsunami 3950 + Millennia X Spectra Physics) producing 5 ps FWHM pulses with an 8.0 MHz pulse repetition rate (3980 Spectra Physics pulse picker). The laser wavelength was selected using a second harmonic generator (LBO crystal, GWN-23PL Spectra Physics) to yield 291 nm excitation pulses directed to a L-format Edinburgh FL900 spectrometer with a monochromator in the emission channel. Single photons were detected by a cooled Hamamatsu R3809U microchannel plate photomultiplier, yielding an instrument response function of ~100 ps. A Soleil-Babinet compensator in the excitation beam and a Glann-Taylor polarizer in the emission beam were used in the anisotropy experiments. The quality of the fit was analyzed based on the reduced-χ^2^ values and the residuals distribution. All the protein samples were prepared in 20 mM Tris HCl pH 8.0 at a concentration of 40 μM.

### Small angle X-ray scattering and modeling

**Figure 1:**
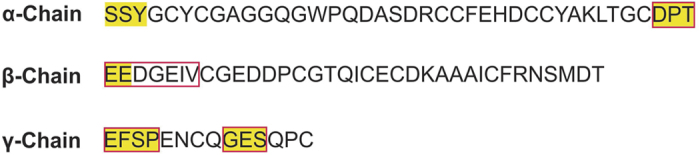
Diagram of crotoxin A (CA) modeling in the CORAL software^43^ based on small angle X-ray data. The amino acid sequences of the three polypeptide chains (α, β and γ) of CA (based on isoform CA_2_ sequence), with the residues not modeled in the CA crystal structure highlighted in yellow and the regions modeled as flexible loops in the CORAL software as red boxes. The three N-terminal residues were not modeled because CORAL requires a minimum of five residues to model loops at the N-terminal portion.

Small angle X-ray scattering (SAXS) experiments were performed with CTX, CA and CB dissolved in 20 mM Tris-HCl pH 8.0 at 10 (±1) mg.ml^−1^ (CA) and 5.0 (±0.5) mg.ml^−1^ at room temperature. The measurements were taken using Bruker-NANOSTAR^TM^, located at the Laboratory of Crystallography at the Institute of Physics of the University of São Paulo. This camera is equipped with a microfocus Genix 3D system (source + focusing mirrors) and two scatterless slit sets for collimation, both provided by Xenocs. The detection is performed by a Vantec-2000 area detector. Scattering experiments on the liquid samples were performed using reusable homemade quartz capillaries glued on stainless steel cases. Background intensities were obtained based on scattering by the corresponding buffers measured using the same capillaries. The data obtained by 3600 s exposure were analyzed using the package SUPERSAXS (Oliveira & Pedersen, unpublished). Experimental data are shown as intensity I(q) versus the momentum transfer *q* = (*4π/λ*)sin *θ*, where *λ* is the radiation wavelength and *2θ* is the scattering angle. After treatment, the data were normalized to an absolute scale using water as the primary standard. The radius of gyration, R_g_, was computed by the indirect Fourier transform method (IFT) using the Gnom package^39^. The pair distances distribution function *p(r*) was also calculated by the IFT method, and the maximum diameter, *D*_*max*_, was obtained. Since the data are normalized to an absolute scale, the molecular mass can be estimated^40^. The SAXS data on CTX, CA and CB were compared with the crystallographic structures (3R0L for CTX crystal structure; 2QOG for CB crystal structure) using the program CRYSOL^41^. As described later, since the CB structure was solved with four monomers in the asymmetric unit, the tetrameric, dimeric and monomeric structures could be tested and compared to the experimental data. *Ab initio* modeling of CB was performed using the program DAMMIN^42^. As the CA crystal structure (PDB ID 3R0L) presents some flexible loops not modeled due to a lack of electron density, these flexible loops were modeled as dummy atoms using the program CORAL^43^ to provide a low-resolution structure for CA. Figure 1 shows the residues not modeled in the crystallographic structure CA and the regions that were modeled using SAXS data. We also modeled four N-terminal residues of the β-chain that are present in the crystal structure as dummy atoms to provide insights on the flexibility of the loops in CA. For CA modeling in CORAL, the α and β-chains were kept fixed, and freedom of movement was permitted for the γ chain, maintaining the disulfide bonds as linkages between the polypeptide chains throughout CORAL refinement. For the structural modeling of CTX, CORAL was used for the CA modeling as explained above and permitted freedom of movement for CB, maintaining Trp36 of CA and Trp30 and Trp61 of CB at the CA/CB interface throughout CORAL refinement. Using CORAL, 10 different models were obtained for CA and CTX, and the program DAMAVER^44^ was used to choose the most representative model of both proteins and generate an averaged envelope (dummy atom model).

## Results

### Composition of CA and CB in *Crotalus durissus terrificus* venom and in CTX complex

The CA and CB composition in three different lots of crude *C. d. terrificus* venom and in the CTX complex purified from these lots were analyzed by integration under the curve of the 280 nm absorbance, showing CA/CB molar ratios of approximately 1.0 in both the crude venoms and their respectively purified CTX samples (Table 1). These data indicate that all CB is complexed with CA at a 1:1 molar ratio in both purified CTX and the crude venom.

### Dynamic light scattering (DLS) studies and PLA_2_ activity assay in reconstituted CTX

Dynamic light scattering (DLS) experiments with CTX indicated a monomodal distribution of the molecules in the sample (>99% mass), with a hydrodynamic radius (R_H_) of 23 Å and a polydispersity of 11.6% (Table 2). This R_H_ value corresponds to a molecular mass (MM) of approximately 23 kDa, which is consistent with a heterodimer formed by a CA (~9.5 kDa) and a CB (~14 kDa) subunit. The isolated CA and CB subunits also presented a unimodal distribution of molecules in the sample (>99% of mass) and R_H_s of 16 Å and 34 Å, with polydispersity values of 15.1% and 7.5%, respectively (Table 2). This R_H_ value obtained for CA corresponds to a MM of approximately 11 kDa (Table 1), which is consistent with a monomer of this subunit. On the other hand, isolated CB displayed the highest value of R_H_ (34 Å) compared with CTX and CA, which corresponds to a MM of approximately 59 kDa (Table 2). This MM is consistent with a tetramer of CB (~54 kDa). The tendency of isolated CB to form oligomers in solution is confirmed by cross-linking assays (Supplementary Fig. 1).

After CA and CB dissociation by RP-HPLC, the CTX complex was reconstituted by mixing isolated CA and CB subunits in a 1:1 molecular ratio to perform DLS experiments in the same previous conditions. The R_H_ and MM values obtained for the reconstituted CTX were similar to the values for native CTX, despite the higher polydispersity obtained in this sample (20.1%) (Table 2). The data also show that the addition of CA to isolated CB samples can disassociate CB tetramers and recover CTX formed by a 1:1 CA/CB molecular ratio. The autocorrelation function and the regularization fit based on the CONTIN algorithm of the measurements are shown in Supplementary Fig. 2.

An enzymatic assay confirmed that PLA_2_ activity remains unaffected in the reconstituted CTX (Supplementary Fig. 3). The linear regression curves for the PLA_2_ activity of native and reconstituted CTX were 3.895*X + 1.651 (R square = 0.9483) and 3.887*X + 1.783 (R square = 0.9146), respectively. The regression slopes that correspond to specific PLA_2_ activities are statistically equivalent (P = 0.9806) (Supplementary Fig. 3).

### Isothermal titration calorimetry (ITC) studies

**Figure 2:**
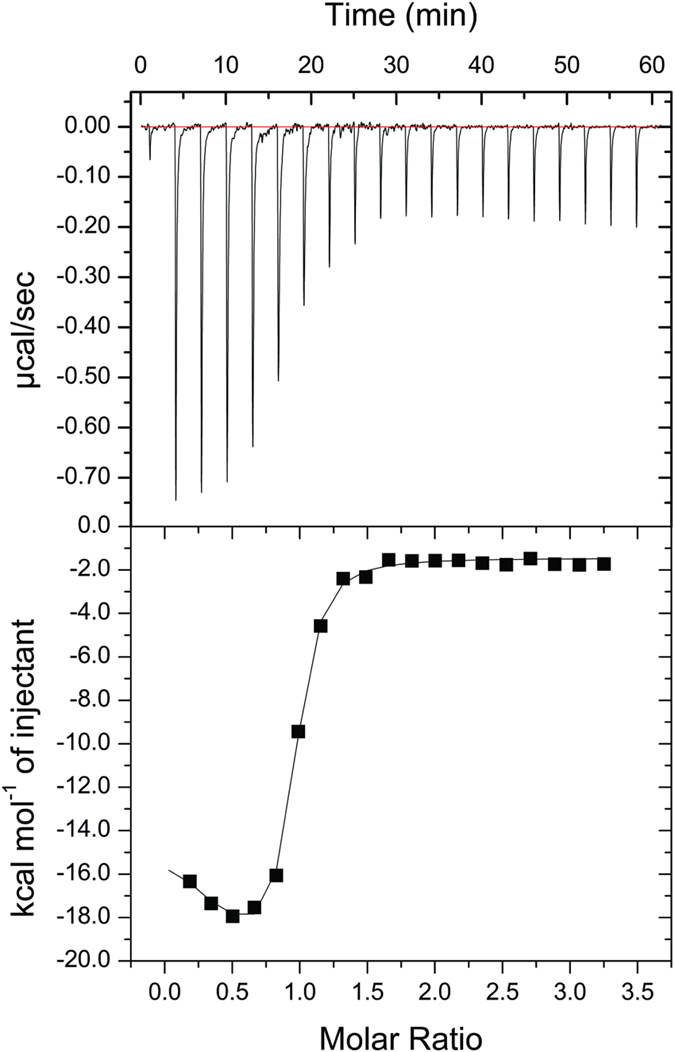
Isothermal titration calorimetry (ITC) of CA (240 μM) into CB (15 μM) in 50 mM ammonium formate (50 mM), pH 6.6, at 25 °C. (Top) Raw data thermogram representing the equipment response (μcal/s) as a function of time (minutes) of the titration and (Bottom) binding isotherm with enthalpy change (kJ/mol of injectant) versus CA-CB molar ratio.

The interactions between CA and CB were assessed by ITC, and a representative calorimetric titration is shown in Fig. 2. Although the thermogram presented a general exothermic binding behavior, the analysis of the integrated peaks showed two different processes. The binding isotherms were fitted with binding polynomials considering two binding events. The dissociation constants and the binding enthalpies are presented in Table 3. Whereas the first event is entropically driven (ΔH > 0; −T*ΔS < 0), the second is enthalpically driven (ΔH < 0; −T*ΔS > 0) (Table 3).

### Steady-state and time-resolved fluorescence spectroscopy measurements

**Figure 3:**
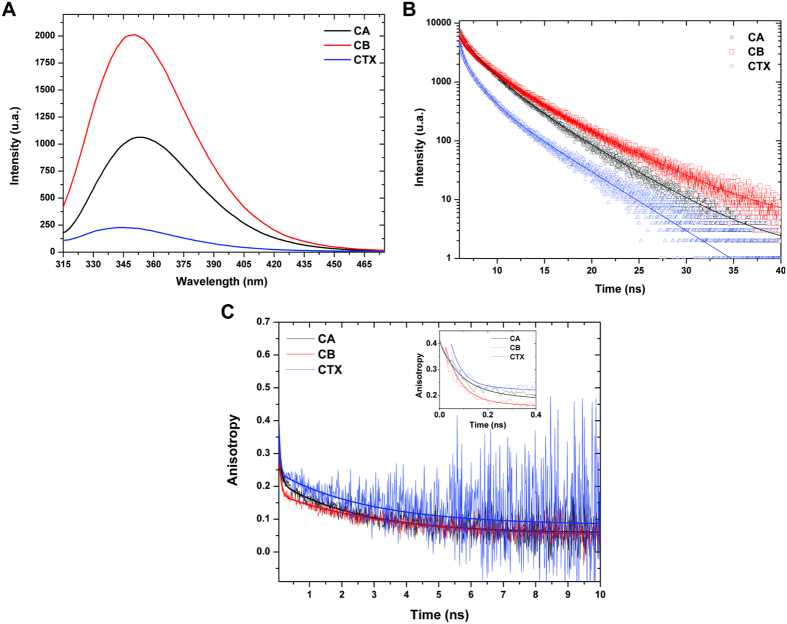
Fluorescence spectroscopy experiments with crotoxin (CTX) and its isolated subunits, crotoxin A (CA) and crotoxin B (CB). (**A**) Steady-state spectroscopy fluorescence of tryptophans from CA (black line); CB (red line) and CTX (blue line). (**B**) Time-resolved fluorescence of tryptophans measured at λ_em_ 360 nm from CA (black line and circles), CB (red line and squares) and CTX (blue line and triangles). (**C**) Time-resolved fluorescence anisotropy measured at λ_em_ 360 nm for tryptophan residues from CA (black), CB (red) and CTX (blue). The inset highlights the first 0.4 nanoseconds from time-resolved fluorescence anisotropy data.

Steady-state fluorescence by the excitation of tryptophan residues showed that isolated CB and CA subunits have higher fluorescence signals than the CTX heterodimer (Fig. 3, panel A). In contrast to CA and CB, which display maximum emission values at 356 and 350 nm, respectively, CTX presents a maximum fluorescence signal at 344 nm (Fig. 3, panel A). These data suggest that the tryptophan residues in the isolated subunits (1 W at CA and 3 W at CB) are in a polar environment, whereas in the CTX heterodimer, they are in a more hydrophobic environment, indicating that all tryptophan residues become hidden after CA and CB complexation for CTX formation. The lifetime fluorescence decays measured at λ_em_ 360 nm highlight this behavior for tryptophans in CTX samples. As usually found in proteins, the intensity decay measured experimentally (Fig. 3, panel B) was best fitted to a tri-exponential curve. A strong reduction in the three tryptophan lifetimes was also observed for CTX in comparison to isolated CA and, especially, isolated CB (Table 4; Fig. 3 panel B). The changes in lifetime are accompanied by a marked decrease in the normalized pre-exponential factor for the long lifetime component and a high value for the short component (Table 4). This phenomenon is also observed for the other emission wavelengths measured (Supplementary Table 1).

Time-resolved fluorescence anisotropy data (Fig. 3, panel C; Table 5) were best fitted to bi-exponential curves. The long rotational correlation time was highest for CB (ϕ_1_ = 3.5 ± 0.2 ns), lowest for CA (ϕ_1_ = 2.5 ± 0.1 ns) and intermediate for CTX (ϕ_1_ = 2.9 ± 0.3 ns; Table 4). These values, which are related to the rotational motion of the whole structure, indicate that CB has a larger structure than CA and CTX, whose low values of ϕ_1_ are consistent with smaller dimensions as observed by DLS and SAXS. Regarding the ϕ_2_ values, which are related to the rotational motion of tryptophans around the bonding to the macromolecule, CTX presented the lowest value (0.047 ± 0.008 ns), CA the highest (0.086 ± 0.006 ns) and CB an intermediate value (0.064 ± 0.003 ns) (Table 5). These differences indicate that the tryptophan side chains in CTX are locally more flexible than in isolated CA and CB subunits.

### Small angle X-ray scattering (SAXS) studies

**Figure 4:**
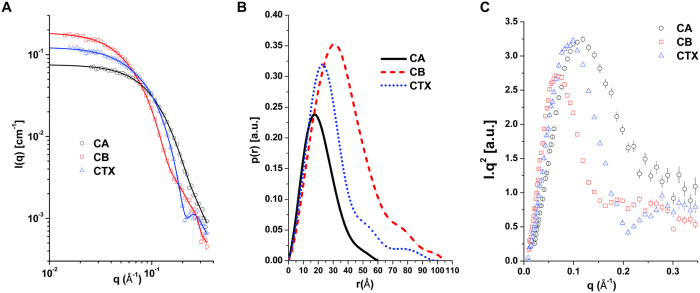
Small angle X-ray scattering curves of crotoxin (CTX) and its isolated subunits, crotoxin A (CA) and crotoxin B (CB). (**A**) Experimental scattering curves of CA (black circles), CB (red squares) and CTX (blue triangles) and scattering curve (straight lines) fitted using GNOM^39^. (**B**) Normalized distance distribution function for CA (black line), CB (red dashes) and CTX (blue dots). (**C**) Krakty plots of SAXS data from CA (black circles), CB (red squares) and CTX (blue triangles).

**Figure 5:**
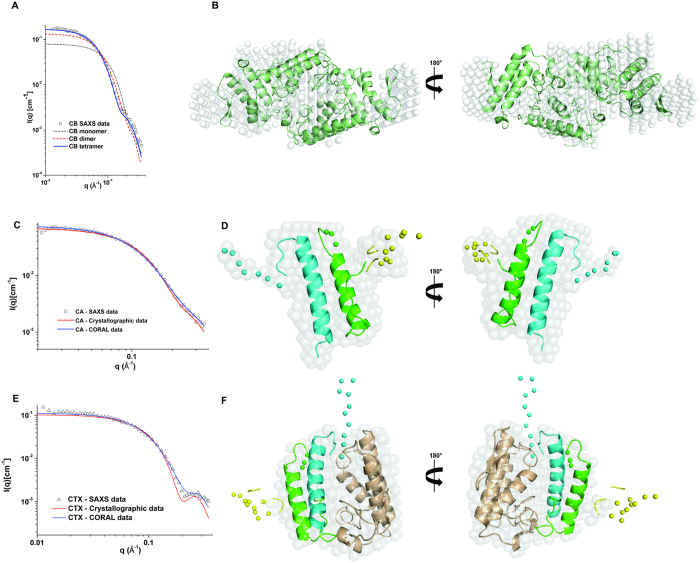
Small angle X-ray scattering models of CTX and CA and comparison of theoretical SAXS curves from crystal and structural models with experimental SAXS curves of crotoxin (CTX), crotoxin A (CA) and crotoxin B (CB). (**A**) Fitting of monomer (black dots), dimer (red dashes) and tetramer (blue line) of crotoxin B to experimental CB SAXS data (black squares). (**B**) Superposition of crystal structure of CB (in green cartoon) on SAXS dummy chain model (white transparent surface) and after 180° rotation. (**C**) Fitting of theoretical SAXS curves from crystallographic data of CA (red line) and SAXS model of CA modeled in CORAL software^43^ with loops that were absent in the CA crystal structure (blue line) in experimental CA SAXS data (black circles). (**D**) Cartoon representation of CA model obtained in CORAL software superposed on its SAXS dummy chain model (white transparent surface) and after 180° rotation. Chains α, β, γ are shown in green, blue and yellow, respectively. The loops modeled as dummy atoms by CORAL software are shown as solid spheres. (**E**) Fitting of theoretical SAXS curves from crystallographic data of CTX (red line) and SAXS model of CTX obtained by CORAL (blue line) from experimental CA SAXS data (black triangles). (**F**) Cartoon representation of CTX model obtained in CORAL superposed on its SAXS dummy chain model (white transparent surface) and after 180° rotation. Chains α, β, γ of CA are shown in green, blue and yellow, respectively. CB is shown in brown. The loops of CA modeled as dummy atoms by CORAL are shown as solid spheres.

The radius of gyration (R_g_) calculated from the p(r) curves and molecular mass determined in the SAXS experiments were, respectively, 23.9 ± 0.2 Å and 36 ± 4 kDa for CTX; 29.6 ± 0.1 Å and 56 ± 8 kDa for CB; and 16.95 ± 0.07 Å and 11 ± 3 kDa for CA (Fig. 4). The R_g_ values obtained by Guinier analysis are similar to the values obtained from the p(r) curves, and the linear behavior of the data in the Guinier region highlights the monodispersity of the samples (Supplementary Fig. 4). These data show that CA has a molecular mass close to expected value (~9.5 kDa as a monomer) and that CB (~14 kDa as monomer) presents a larger R_g_ and molecular mass than CTX (~23 kDa), suggesting that isolated CB may form larger oligomers than CTX. The tetrameric crystal structure of CB isolated from *Crotalus durissus terrificus* venom (PDB ID 2QOG) provided a good fit to the scattering data, compared to the monomeric and dimeric forms (Fig. 5, panel A). In fact, the superposition between the CB crystal structure and *ab initio* dummy model presents satisfactory agreement (Fig. 5, panel B).

The monomeric structure of the CA obtained from the CTX crystal structure (PDB ID 3R0L) provides a relatively good fit to the scattering data (Fig. 5, panel C). However, the crystallographic CA structure was modeled without some residues located on flexible loops due to an absence of electron density^5^. Thus, the majority of missing loops were modeled by using CORAL software, which models the loops by fitting the SAXS data. Figure 1 shows the residues that were not modeled in the crystal structure and the regions modeled as dummy atoms using the SAXS data. To gain insights into the loop flexibility of CA, the four N-terminal residues of the β chain were also modeled using SAXS data (Fig. 1). The resulting model shows a slight improvement in the fit of the scattering data (Fig. 5, panel C) compared to the crystallographic CA structure.

Finally, the CTX crystal structure does not provide a good fit to the scattering data (Fig. 5, panel E). Thus, CTX was modeled by the same procedure for CA reconstruction, using the CORAL software with SAXS data. In addition, based on fluorescence spectroscopy data (see section 3.4) and tryptophan contacts in the CA/CB interface in the CTX crystal structure (PDB ID 3R0L), the tryptophan residues (Trp36 of CA, Trp31 and Trp70 of CB) were kept in the CA/CB interface when constructing the CTX SAXS. The best model presents a better fit to the scattering data than the crystal structure model (Fig. 5, panel E). The Krakty plots (Fig. 4, panel C) show that the SAXS models exhibit a well-defined conformation and little flexibility.

## Discussion

Crotoxin was the first animal toxin to be purified and crystallized, with its first crystallization report dated 1938^45^. However, the first crystal structures of CTX and its isolated subunits were solved only recently, despite two previous X-ray diffraction reports^46^,^47^. This difficulty, as previously noted^5^, can be attributed to the large number of CTX isoforms, which impairs the ability to obtain the homogenous sample required for crystals to produce high-quality X-ray diffraction patterns.

Despite these crystallization attempts, several other authors have tried to obtain structural information on CTX by solution biophysical methods, such as small angle X-ray scattering, fluorescence spectroscopy and circular dichroism^48^,^49^. Here, we used these techniques allied to other ones (DLS, ITC and time-resolved fluorescence spectroscopy), combining these biophysical data with interpretation of the now available CTX and CB crystal structures (PDB IDs 3R0L and 2QOG, respectively).

The first small angle X-ray scattering analysis performed with CTX, CA and CB showed a R_g_ of 16.5 Å, 12.4 Å and 32.4 Å for CTX, CA and CB, respectively^48^. Despite the differences in R_g_ observed in our experiments (23.9 Å, 16.9 Å and 29.6 Å CTX, CA and CB, respectively), both data indicated a larger size for isolated CB than CTX in solution. Our DLS data show compatible sizes of R_h_ with the R_g_ values obtained from the SAXS data (R_h_ of 23 Å, 16 Å and 34 Å CTX, CA and CB, respectively), highlighting this size difference between CB and CTX. These data suggest that CB may form oligomers since whereas CB as a monomer is ~14 kDa, CTX is ~23 kDa. In fact, CB has previously been observed as dimers and tetramers by DLS and crystallography^33^, and we highlighted the oligomerization of CB in this work through cross-linking assays, DLS measurements and SAXS data.

Previously, a stronger fluorescence signal was observed in isolated subunits compared to intact CTX, similarly to the data presented here^49^. However, these authors attributed the result to artifacts resulting from irreversible structural changes that occur upon subunit isolation by urea ion-exchange chromatography. Here, we discard this hypothesis, since we isolated the CA and CB subunits in the absence of urea, and our samples provided typical CD spectra of alpha-helical rich-proteins, as expected for phospholipases A_2_ (Supplementary Fig. 5).

**Figure 6:**
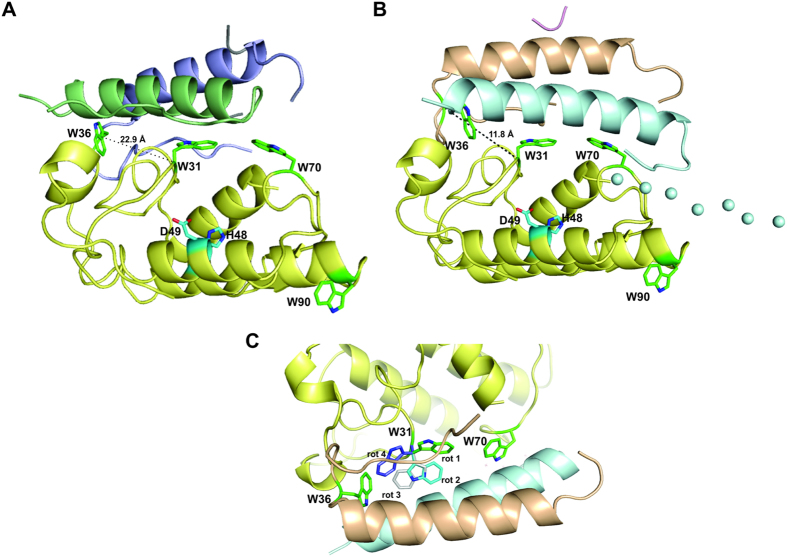
Structural studies of tryptophan residues at CA/CB interface in CTX heterodimer. (**A**) Cartoon representation of crystal structure of CTX (PDB ID 3R0L). Chains α and β from CA are shown in blue and green, respectively. CB is shown in yellow. (**B**) Cartoon representation of SAXS model of CTX. Chains α, β, γ of CA are shown in wheat, blue, and purple, respectively. CB is shown in yellow. The loops of CA modeled as dummy atoms by CORAL software^43^ are shown as solid spheres. The tryptophan residues of CA (Trp36) and CB (Trp31, Trp70 and Trp90) are highlighted as green sticks. Residues of the catalytic site of CB (His48 and Asp49) are highlighted as cyan sticks. C_α_ distance between Trp36 of CA and Trp31 from CB is shown in black dashes. (**C**) A rotation of 180° of the cartoon representation of the SAXS model of CTX illustrated in panel B shows the possible rotamers of Trp31 from CB. Rotamer 1 represents the tryptophan rotamer presented by the CTX crystal structure, whereas other rotamers (2, 3 and 4) are obtained by the analysis of rotamer possibilities in the Coot software^74^. According to this software, rotamers 1, 2, 3 and 4 are present in 32%, 18%, 16% and 11% of the crystallographic structures available in the PDB.

The CTX crystal structure (PDB ID 3R0L) indicated that Trp31 and Trp70 of the CB subunit lie in the interface with the β-chain of CA and contribute to the stability of the CTX heterodimer, with Trp31 completely buried in the heterodimer interface and Trp70 CB partially exposed to the solvent (Fig. 6, panel A)^5^. In contrast, Trp90 of CB is not in contact with CA and is completely exposed to the solvent (Fig. 6, panel A). Trp36 of CA, which is located on its α chain and at the CA/CB interface, is partially exposed to the solvent (Fig. 6, panel A).

The spectroscopy fluorescence data provided here suggest that all tryptophans (Trp36 from CA, Trp31, Trp70 and Trp90 from CB) become hidden in the CTX heterodimer structure. However, because 3 of 4 tryptophan residues in the CTX crystal structure are partially or totally exposed to the solvent (Fig. 6, panel A), the spectroscopy fluorescence data are not in agreement with the crystallographic model. On the other hand, we obtained a SAXS model that presents a better fit to the SAXS data than the crystal structure, where Trp31 from CA, Trp36 and Trp70 from CB are completely buried in the inner CTX structure (Fig. 6, panel B). Besides, Trp90 from CB is partially occluded by a disordered loop in the N-terminal region of the β-chain from CA (Fig. 6, panel B).

The time-resolved fluorescence spectroscopy data suggest the occurrence of complex processes caused by the approximation of tryptophan from CA and CB in the CTX heterodimer. The reduction in fluorescence intensity is consistent with the decrease in the fluorescence lifetimes of the tryptophans in CTX and the increased contribution from the pre-exponential factor corresponding to the short lifetime component. An increase in the fluorescence emission of tryptophans immersed in hydrophobic regions would be expected; however, quenching from nearby residues in the CTX heterodimer can promote non-radiative pathways for the de-excitation of the fluorophores. Trp36 of CB is the closest tryptophan residue from the CB structure to Trp31 of CA in the crystallographic and SAXS structural models (Fig. 6). The analysis of the distance between these residues in both structural models shows that the Trp36CA/Trp31CB Cα distance in the SAXS model is shorter than in the crystallographic model (11.8 Å and 22.9 Å, respectively) (Fig. 6, panels A and B). The analysis of the rotamers of Trp31 in CB shows that its side chain can lie even closer to Trp36 from CA (Fig. 6, panel C). It has been proposed that the multi-exponential decay of Trp in peptides and proteins results from the occurrence of different rotamers and that the pre-exponential factors are related to the relative rotamer populations^50^,^51^. Our observation of changes in pre-exponential factors due to CTX heterodimer formation is then related to the conformational changes affecting the Trp residues. In addition, time-resolved anisotropy fluorescence shows that CTX presents the lowest ϕ_2_ values (Table 5). This result may be due to the occurrence of homotransfer between Trp residues caused by the reduction of the distances in the formation of the CTX heterodimer. Beyond the kinetic parameters, it is wise to pay attention to the structural parameter represented by the residual anisotropy. It reflects structural restrictions on the rotation of the emission dipole and is the main contribution to the steady state anisotropy^52^. Its higher values for CTX (Table 5) show that, despite the flexibility of the tryptophan inside the protein, they are not entirely free to rotate.

These data suggest that the SAXS model is in better agreement with the fluorescence spectroscopy data than the crystallographic model. However, the differences observed between the crystallographic and SAXS models of CTX could result from the different protein samples used to perform the experiments. The authors of crystal structure crystallized and solved the structure of a single isoform of crotoxin (CA_2_CB_b_; class I isoform)^5^, whereas in this work, we used a natural pool of the isoforms of CTX, CA and CB. Thus, the interface of CA/CB may vary between different isoforms. However, it is important to note that fluorescence spectroscopy data using the natural pool of isoforms supports that, independent of isoform, all tryptophans seem to be occluded in the CTX structure, which is especially notable since the Trp31 and Trp70 residues lie at the entrance of the catalytic site of CB (Fig. 6).

**Figure 7:**
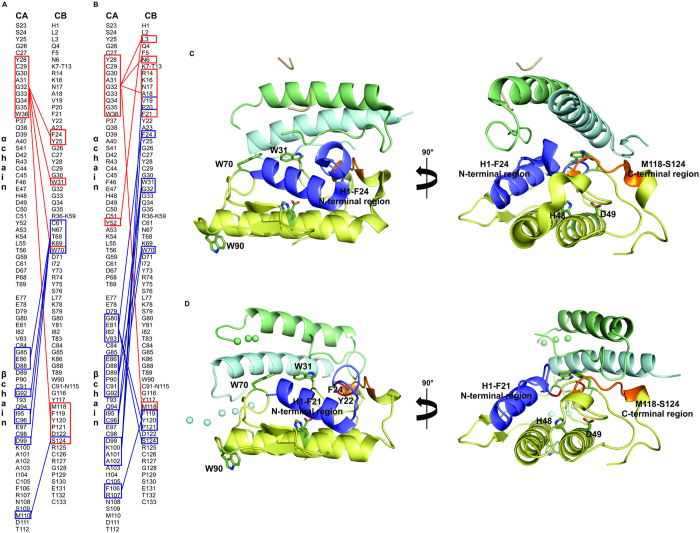
Structural comparisons of CA/CB interface of CTX crystallographic and SAXS structural models. (**A**) Diagram of interactions between α-chain (red boxes) and β-chain of CA (blue boxes) with CB in CTX SAXS model obtained using PSAIA software^53^. (**B**) Diagram of interactions between α-chain (red boxes) and β-chain of CA (blue boxes) with CB in CTX crystal structure obtained using PSAIA software^53^. (**C**) Cartoon representation of crystal structure of CTX (PDB ID 3R0L) and after 90° rotation highlighting the His1-Phe24 N-terminal region (in blue) and Met118-Ser124 C-terminal region (in orange) of CB (yellow). Chains α, β, γ of CA are shown in green, blue, and wheat, respectively. Trp31, Trp70, Trp90 and active site residues His48 and Asp49 from CB are highlighted in dark green sticks. (**D**) Cartoon representation of CTX SAXS model after 90° rotation, highlighting the His1-Phe21 N-terminal region (in blue) and the Met118-Ser124 C-terminal region (in orange) of CB (yellow). C^α^ of Tyr22 and Phe24 are highlighted in red. Chains α and β of CA are shown in green and blue. Trp31, Trp70, Trp90 and active site residues His48 and Asp49 of CB are highlighted as dark green sticks. The loops of CA modeled as dummy atoms by CORAL software are shown as solid spheres.

Analysis of the CA/CB interface at the crystallographic and SAXS models using the PSAIA software^53^ shows that the physical-chemical nature of the residues in the CA/CB interface are similar in both models (Fig. 7). This analysis shows that γ-chain does not make any contacts with CB in either structural model. Besides, it shows that both models present similar regions of the α and β-chains of CA that are in contact with CB, but in a different way (Fig. 7). The 24–25, 31–33 and C-terminal regions of CB are in contact with CA in both structural models; however, whereas these regions of CB are in contact with the β-chain of CA in the CTX crystal structure, in the CTX SAXS model these regions are in contact with the α chain of CA (Fig. 7). The Trp70 residue in the CTX crystal structure interacts with the β-chain of CA, whereas in the CTX SAXS model, this residue interacts with both the α and β-chains of CA, with the β-chain in contact with a large portion around this residue (61–70) exclusively in the SAXS model. In contrast, the interaction of the N-terminal region of CB with the α-chain of CA is observed only in the CTX crystal structure. In the SAXS model, the majority of the N-terminal portion is exposed to the solvent (Fig. 7).

**Figure 8:**
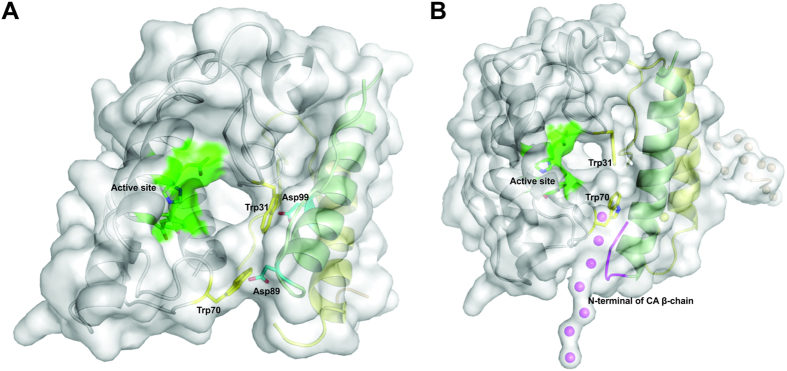
Accessibility of catalytic site from CB in CTX crystallographic and SAXS structural models. (**A**) Cartoon representation of CTX crystal structure (PDB ID 3R0L) covered by white surface, where chains α, β, γ of CA are shown in green, yellow and wheat, respectively. CB is shown as a white cartoon. Trp31 and Trp70 residues from CB_b_ isoform are highlighted by yellow sticks. The active site of CB (His48, Asp49, Tyr53 and Asp99) are represented in green. Asp89 and Asp99 residues of CA are shown as cyan sticks. CTX crystal structure is formed by a CA and a CB isoform from class I (CA_2_CB_b_ isoforms). Despite partial blocking of the active site by Trp31 and Trp70 residues from the CB_b_ isoform in a front view (residues that establish hydrogen bonds with Asp89 and Asp99 with β-chain of CA), it is possible to observe access to the catalytic site from a lateral view. (**B**) Cartoon representation of SAXS model covered by white surface where chains α, β, γ of CA are shown in green, yellow and wheat, respectively. CB is shown as a white cartoon. Trp31 and Trp70 residues from CB are highlighted by yellow sticks. Active site of CB (His48, Asp49, Tyr53 and Asp99) is represented in green. The disordered N-terminal region of β chain from CA (highlighted in magenta) is close to the accessible pocket of the catalytic site in this SAXS model. Since this region is very flexible, it can adopt different structural positions in crotoxin isoforms I and II, possibly contributing to catalytic site blocking in class I isoforms.

The crystal structures of CTX and CB show that Trp31 and Trp70 from CB lie in the entrance of the catalytic site from this phospholipase A_2_ (Figs 6 and 8)^5^,^33^. His48 and Asp49, the main residues involved in the nucleophilic attack and coordination of cofactor Ca^2+^, form the catalytic site of this class of proteins. Hydrogen bonds established with Tyr53 and Asp99 help to stabilize the His48 atoms involved in the nucleophilic attack^54^,^55^,^56^,^57^. CA does not exhibit catalytic activity; however, it inhibits the catalytic activity of CB, particularly from class I isoforms^32^,^58^. Thus, the fact that Trp31 and Trp70 from CB are in contact with the CA chains in the crystallographic and SAXS structural models (Figs 6 and 8) could explain the impact of CA on the catalytic activity of CB.

Previous analysis of the crystal structure of a class I CTX (CA_2_CB_b_ isoforms) shows that the Asp89 and Asp99 residues from the β-chain of the CA establish hydrogen bonds with the Trp31 and Trp70 residues from CB_b_, causing a partial blocking of the CB_b_ catalytic site^5^ (Fig. 8, panel A). A lateral view of the CTX crystallographic heterodimer, however, shows that is it still possible to access the pocket of the catalytic site that is not totally blocked by CA (Fig. 8, panel A). In fact, this crystal structure includes an acetate ion that establishes hydrogen bonds with His48 and Asp49 and can occupy the position of the free fatty acid after the hydrolysis of *sn*-2 acyl groups^5^. The presence of this acetate ion highlights the active site accessibility in this CTX crystal structure model.

Regarding the accessibility of the active site in the SAXS model proposed here, it is difficult to compare the structural differences between class I and class II isoforms because a natural pool of isoforms of CA, CB and CTX was used. In this SAXS structural model, the catalytic site of the CB is partially blocked by CA, whose α and β-chains establish contacts with Trp31 and Trp70 from CB (Figs 7 and 8). However, the CB active site is still accessible from a lateral direction, similarly to the CTX class I crystal structure (Fig. 8, panel B). Thus, the crystallographic and SAXS structural models suggest that the catalytic site of CB is still partially accessible in the CTX heterodimer, which could explain the catalytic activity of CTX heterodimer observed here for native and reconstituted CTX (Supplementary Fig. 3) and in several previously biochemical experiments^32^,^58^. Although the active site of CB is still accessible, the partial blocking by CA is sufficient to prevent the alkylation of the His48 residue of CB on the CTX heterodimer by *p-*bromophenacyl bromide (BPB), a classical irreversible phospholipase A_2_ inhibitor^58^.

Analysis of the SAXS model shows that the disordered N-terminal region of the β-chain from CA is close to the accessible pocket of the catalytic site in this structural model. Since this region is very flexible, it can adopt different structural positions for the several combinations of CA and CB isoforms and may interfere with the accessibility of the catalytic site. This possibility could help to justify the different levels of catalytic activity observed between CTX isoforms.

The crystal structure of isolated CB from *Crotalus durissus terrificus* suggested that in the absence of CA, CB can form tetramers^33^. This crystal structure is formed by two dimers, each dimer consisting of a CBc and a CBa_2_ isoform. Since these isoforms are from class I and class II, respectively, the crystal structure suggests that dimers of CB can be formed by mixture of isoforms from the two different classes. In addition, these authors observed the formation of dimers and tetramers by DLS and non-reduced SDS-PAGE analyses. Remarkably, dimers of CB have also been observed by reduced SDS-PAGE^33^. The cross-linking assays, DLS and SAXS data provided here highlight the tetrameric formation of CB in the absence of CA. Moreover, we demonstrated that the crystallographic model of CB fits the SAXS data well, indicating that this oligomeric structure is also observed in solution. Furthermore, preliminary X-ray data analysis of the CB dimer from *Crotalus durissus collineatus* suggest that this dimer also consists of two different CB isoforms^59^.

It was previously demonstrated that isolated CA and CB components could associate spontaneously in a 1:1 molecular ratio to reconstitute the CTX complex^58^. Furthermore, it was demonstrated that when CB associates with CA, it totally loses its ability to form oligomers^58^. Here, we highlighted this spontaneous association of CA and CB to form CTX (and the consequent dissociation of the CB oligomer) by DLS, since the mixture of CA + CB isolated subunits (reconstituted CTX) has the same R_H_ as intact CTX (Table 2). Remarkably, reconstituted CTX has similar enzymatic activity to native CTX (Supplementary Fig. 3). Moreover, the composition of CTX, CA and CB in crude venom was analyzed, and the results showed no free CB and CA in the venom, with all CB complexed to CA in a 1:1 molar ratio (Table 1).

The isothermal titration calorimetry (ITC) assays performed here between CA and CB showed the occurrence of two different processes (Fig. 2; Table 3) that are characteristic of allostery, cooperativity or conformational changes^60^,^61^. Based on the results obtained by DLS and previous data that showed CA to impair the CB oligomerization^58^, we assign the first event to the dissociation of the CB oligomer and the second event to the formation of the CTX heterodimer. Both events presented dissociation constants in the submicromolar range (Table 3). The first event is entropically driven (ΔH > 0; −T*ΔS < 0; Table 3), related to a favorable conformational entropy caused by an increase in the number of accessible conformations^62^, which is consistent with CB oligomer dissociation. The second event is enthalpically driven (ΔH < 0; −T*ΔS > 0; Table 3) and may result from the formation of van der Waals interactions, hydrogen bonds and electrostatic interactions^63^, which is consistent with the formation of the CTX heterodimer. Therefore, the ITC data highlight the ability of CA to dissociate CB oligomer to reconstitute the CTX complex.

It was previously suggested that CB oligomers is the most catalytic active state of this protein, because a reduction in catalytic activity was observed in the monomeric conformation at lower pHs^58^. Remarkably, the active state of β-neurotoxin from *Crotalus atrox* is a dimer^64^, and ammodytoxin (AtxA), a β-neurotoxin from *Vipera ammodytes ammodytes,* can also form dimers^30^. Based on the crystal structures of isolated CB and AtxA, it was proposed that the oligomeric association of these proteins may increase their neurotoxicity through the creation of new binding sites^30^,^33^. In fact, both CB and ammodytoxin can have similar binding sites at presynaptic membranes since that isolated CB, was able to completely inhibit the binding activity of radioiodinated ammodytoxin^26^.

However, in the interaction of CTX with crotoxin protein acceptor (CAPT) isolated from *Torpedo marmorata* presynaptic membranes, CA remains attached to CB-CAPT, forming a CA-CB-CAPT ternary complex^24^. Moreover, CA enhances the blocking of the neuromuscular transmission of CB^4^,^65^,^66^, potentiates the muscle necrosis caused by CB in rat skeletal muscle^17^ and decreases the adsorption of CB to non-saturable binding sites, thereby restricting its binding to critical target sites at neuromuscular junctions^7^,^8^.

In this context, it remains a challenge to understand the mechanism of action of CTX neurotoxicity, since CA potentiates CB activity in the muscle and at presynaptic membranes in the neuromuscular junction; however, at the same time, it impairs oligomer formation and reduces the catalytic activity of CB through partial blocking of its active site. These observations suggest that other regions of CB may be involved in neurotoxicity in addition to the catalytic activity. In fact, it was demonstrated that the C-terminal region of CB could be involved in the toxicity of CTX, since antibodies against the C-terminal part of AtxA bound to the C-terminal peptides of CB, protecting mice against the lethal potency of CB^28^. Finally, the N-terminal region of AtxA may be involved in the neurotoxicity mechanism, especially the regions Met7-Gly11 and the aromatic Phe24^67^,^68^.

The predicted interfacial binding surface (IBS) of CB includes several residues from the N-terminal portion: Leu2, Leu3, Lys7, Lys10, Ala18, Val19, Ala23 and Phe24^69^,^70^. Remarkably, it was indicated that chemical modification of Tyr22 from CB reduced its neurotoxicity and binding affinity for presynaptic membranes^27^. Finally, peptide-array analysis showed that the N-terminal region of CB (Phe11-Ala18) could constitute a pharmacological site of this protein^71^. Thus, as CA enhances the toxicity of CB at the neuromuscular junction, targeting CB to the target sites, it is possible to suggest that in the CTX heterodimer, there is a region of CB that is not in contact with CA but exposed to the solvent and able to interact with targets. However, in the CTX crystal structure, all above-mentioned regions that would be involved in CTX neurotoxicity (N-terminal, C-terminal and active site regions) are completely hidden in the CA/CB interface (Fig. 7). In contrast, in the SAXS structural model, the N-terminal region (His1-Phe21) is not in contact with CA and is exposed to the solvent (Fig. 7). Therefore, combining our structural data with previous biochemical data from the literature, we propose that the N-terminal region of CB could be the first binding site of CTX at the target sites. After CB N-terminal binding to the target, CA would dissociate from CB, allowing the interaction of the C-terminal of CB with the target as well as making the catalytic site of CB totally accessible. It is relevant to note that CA also occludes Tyr22 and Phe24 in the crystallographic and SAXS structural models and would interact with the membrane only after CA dissociation (Fig. 7).

This hypothesis suggests that the different regions of CB are involved in the expression of neurotoxicity, similarly to a suggestion previously made for AtxA^72^. Additionally, the involvement of the N- and C-terminal regions in the expression of toxicity is also observed on myotoxic PLA_2_-like proteins^73^.

## Concluding remarks

Here, significant information is provided on the interfaces between the CA and CB subunits of CTX, especially regarding the role of tryptophans in the CA/CB interface. The static and time-resolved spectroscopy fluorescence data suggest that all four tryptophan residues of the CTX heterodimer lie in the CA/CB interface. The SAXS structural model shows that Trp36 from CA and Trp31 and Trp70 from CB are particularly important for CA/CB interaction, causing a partial blocking of the CB catalytic site. Furthermore, based on this new structural model, it was possible to suggest a mechanism of action for the toxicity of CTX, where the N-terminal region of CB could constitute the first binding site of CTX to the target, before CA dissociation. Engineered protein mutants are necessary to confirm this hypothesis, but the combination of this structural information with calorimetric data on the CA/CB interaction may be useful for the structure-based design of antineurotoxic inhibitors. Moreover, because CTX displays immunomodulatory, anti-inflammatory and analgesic activities, detailed information on its tertiary and quaternary structures will be very useful for understanding these effects. Finally, as CTX presents anti-tumor activities, including the inhibition of tumor growth, myotoxicity in tumor cells and the induction of apoptosis^10^, structural information could be important for the design of new chemotherapeutic agents.

## Additional Information

**How to cite this article**: Fernandes, C. A. H. *et al*. Biophysical studies suggest a new structural arrangement of crotoxin and provide insights into its toxic mechanism. *Sci. Rep.* **7**, 43885; doi: 10.1038/srep43885 (2017).

**Publisher's note:** Springer Nature remains neutral with regard to jurisdictional claims in published maps and institutional affiliations.

## Supplementary Material

Supplementary Information

## Figures and Tables

**Table 1:** CA/CB molar ratio in three different lots [A: white variety from Queluzito; B: yellow variety from Carrancas; C: reference venom from FUNED used in anti-crotalic serum production (white)] of *Crotalus durissus terrificus* crude venom and in their respectively purified CTX.

Venom identification	Concentration (μM)					
	CA		CB		Molar ratio CB/CA	
	Venom	CTX	Venom	CTX	Venom	CTX
A	9.95	12.00	10.91	11.35	1.10	1.04
B	89.86	15.17	77.86	15.73	0.87	1.04
C	22.10	29.53	20.17	28.48	0.91	0.96
Mean					0.96	1.01

The molar ratio was calculated by 280 nm absorbance under curve integration after reversed phase chromatography. Based on the average amino acid compositions of the CA and CB isoforms, molar extinction coefficients at 280 nm (12,761 cm^−1^M^−1^ and 32,190 cm^−1^M^−1^, respectively) were used in the calculations.

**Table 2:** Hydrodynamic radius (R_H_), polydispersity percentage and molecular mass obtained by dynamic light scattering (DLS) experiments.

	R_H_ (Å)	MW (kDa)	% Pd	% Mass
CA	16	11	16.1	99.7
CB	34	59	7.5	99.6
CTX	23	23	11.9	99.4
CA + CB	22	21	20.1	99.6

The percent mass represents the amount of mass of the molecule with the hydrodynamic radius obtained. CA + CB samples were obtained by mixture of the isolated subunits at a 1:1 molecular ratio.

**Table 3:** Thermodynamic parameters for CA-CB binding obtained by isothermal titration calorimetry (ITC).

	K_d_ (μM)	ΔH (kcal.mol^−1^)	−T.ΔS (cal.mol^−1^.deg^−1^)
Event 1	0.11 ± 0.05	10.52 ± 2.36	−67.0
Event 2	0.16 ± 0.04	−26.03 ± 5.23	56.1

**Table 4:** Lifetimes (t) and respective pre-exponential factors (α) of tryptophan fluorescence emission at 306 nm of CA, CB and CTX obtained by time-resolved spectroscopy fluorescence.

	CA	CB	CTX
t_1_ (ns)	4.32 ± 0.03	4.96 ± 0.03	4.06 ± 0.04
t_2_ (ns)	1.68 ± 0.03	1.65 ± 0.02	1.22 ± 0.02
t_3_ (ns)	0.35 ± 0.01	0.19 ± 0.01	0.16 ± 0.03
α_1_	0.19 ± 0.01	0.19 ± 0.01	0.06 ± 0.02
α_2_	0.43 ± 0.01	0.31 ± 0.01	0.21 ± 0.01
α_3_	0.38 ± 0.01	0.50 ± 0.04	0.72 ± 0.07
χ^2^	1.18	1.11	1.13

The quality of the curve fit shown in Fig. 3, panel B analyzed based on the reduced-χ^2^ is also shown.

**Table 5:** Lifetimes (ϕ) and respective pre-exponential factors (α) of time-resolved anisotropy fluorescence at 360 nm of tryptophans from CA, CB and CTX.

	CA	CB	CTX
φ_1_ (ns)	2.5 ± 0.1	3.5 ± 0.2	2.9 ± 0.3
φ_2_ (ns)	0.086 ± 0.006	0.064 ± 0.003	0.047 ± 0.008
α_1_	0.156 ± 0.003	0.129 ± 0.002	0.158 ± 0.006
α_2_	0.225 ± 0.014	0.257 ± 0.014	0.205 ± 0.043
A_res_	0.034	0.046	0.082
χ^2^	1.07	1.11	1.06

The quality of the curve fit shown in Fig. 3, panel C analyzed based on the reduced-χ^2^ is also shown. The residual anisotropy (A_res_) of each sample is also indicated.
