# Supplementary material for: Biophysical studies suggest a new structural arrangement of crotoxin and provide insights into its toxic mechanism
Source: Sci Rep. 2017 Mar 3;7:43885. doi: 10.1038/srep43885 (PMC5335569; doi:10.1038/srep43885)
Supplement: Supplementary Information [file srep43885-s1.pdf]

Biophysical studies suggest a new structural arrangement of crotoxin and provide insights into its toxic mechanism

Carlos A. H. Fernandes[1], Wallance M. Pazin[2], Thiago R. Dreyer[1], Renata N. Bicev[3], Walter L. G. Cavalcante[1,4], Consuelo L. Fortes-Dias[5], Amando S. Ito[2], Cristiano L. P. Oliveira[3], Roberto Morato Fernandez[1];Marcos R. M. Fontes[1*]

[1]Departamento de Física e Biofísica, Instituto de Biociências, Universidade Estadual Paulista, UNESP, Botucatu-SP, Brazil; [2]Departamento de Física, Faculdade de Filosofia Ciências e Letras de Ribeirão Preto, USP, Ribeirão Preto-SP, Brazil; [3]Departamento de Física Experimental, Instituto de Física, Universidade de São Paulo – USP, São Paulo, SP, Brazil; [4]Departamento de Farmacologia, Instituto de Ciências Biológicas, UFMG, Belo Horizonte, MG, Brazil; [5]Diretoria de Pesquisa e Desenvolvimento, Fundação Ezequiel Dias (FUNED), Belo Horizonte, MG, Brazil

[*]Address for correspondence

Dep. de Física e Biofísica, IBB, UNESP, 18618-970, Botucatu/SP, Brazil.
fontes@ibb.unesp.br

Supplementary material

Supplementary Fig. 1. SDS-PAGE (8-25% Phast gels) of CB after cross-linking. Glutaraldehyde concentrations are indicatedat the top and molecular markers (kDa) on the left side of the figure. A and B show different preparations of CB and C shows CTX.

Supplementary Fig. 2. Autocorrelation function and regularization fit based on CONTIN algorithm of (A) CTX; (B) CA; (C) CB and (D) reconstituted CTX by mixing isolated CA and CB subunits at a 1:1 molecular ratio.

Supplementary Fig. 3. Linear regression curves of PLA_2 activity of native (white circles) and reconstituted (by mixing CA and CB isolated subunits)CTX (black circles). The regression curves were 3.895*X + 1.651 for native CTX with R squared of 0.9843 and 3.887*X + 1.783 for reconstituted CTX with R squared of 0.9146. A pooled slope of 3.8905 was generated when both activity curves were analyzed together.

Supplementary Fig. 4. Guinier plot and radius of gyration (R_g) calculated by Guinier analysis from small angle x-ray scattering data on (A) CA; (B) CB and; (C) CTX.

Supplementary Fig. 5. Far-UV circular dichroism spectra of CTX (blue line), CA (black line) and CB (red line).

Supplementary Table 1. Mean lifetimes (ns) of total tryptophan fluorescence emission at different wavelengths of CA, CB and CTX obtained by time-resolved spectroscopy fluorescence.

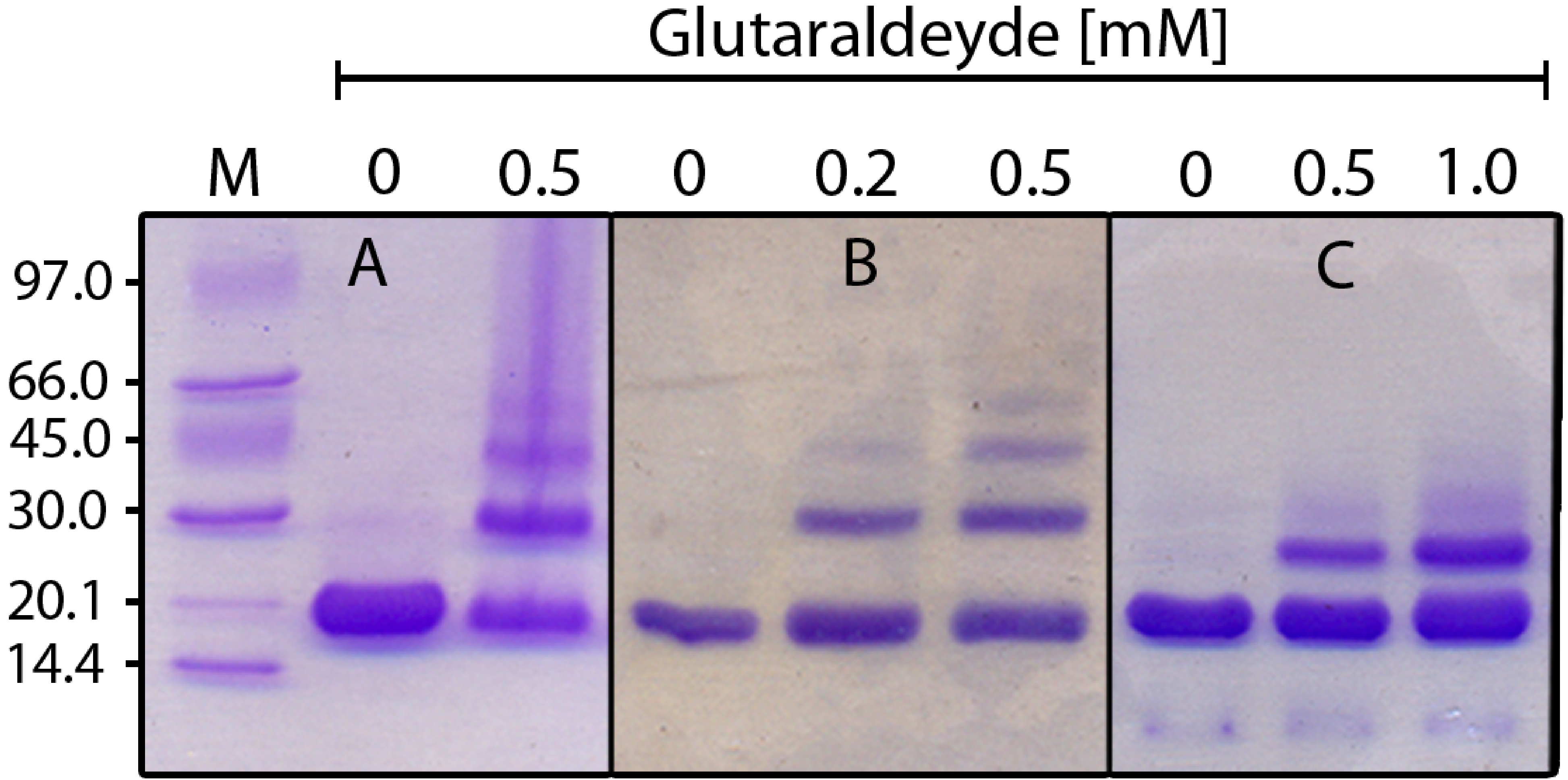
Glutaraldeyde [mM]
M
0
0.5
0
0.2
0.5
0
0.5
1.0
A
B
C
97.0
66.0
45.0
30.0
20.1
14.4

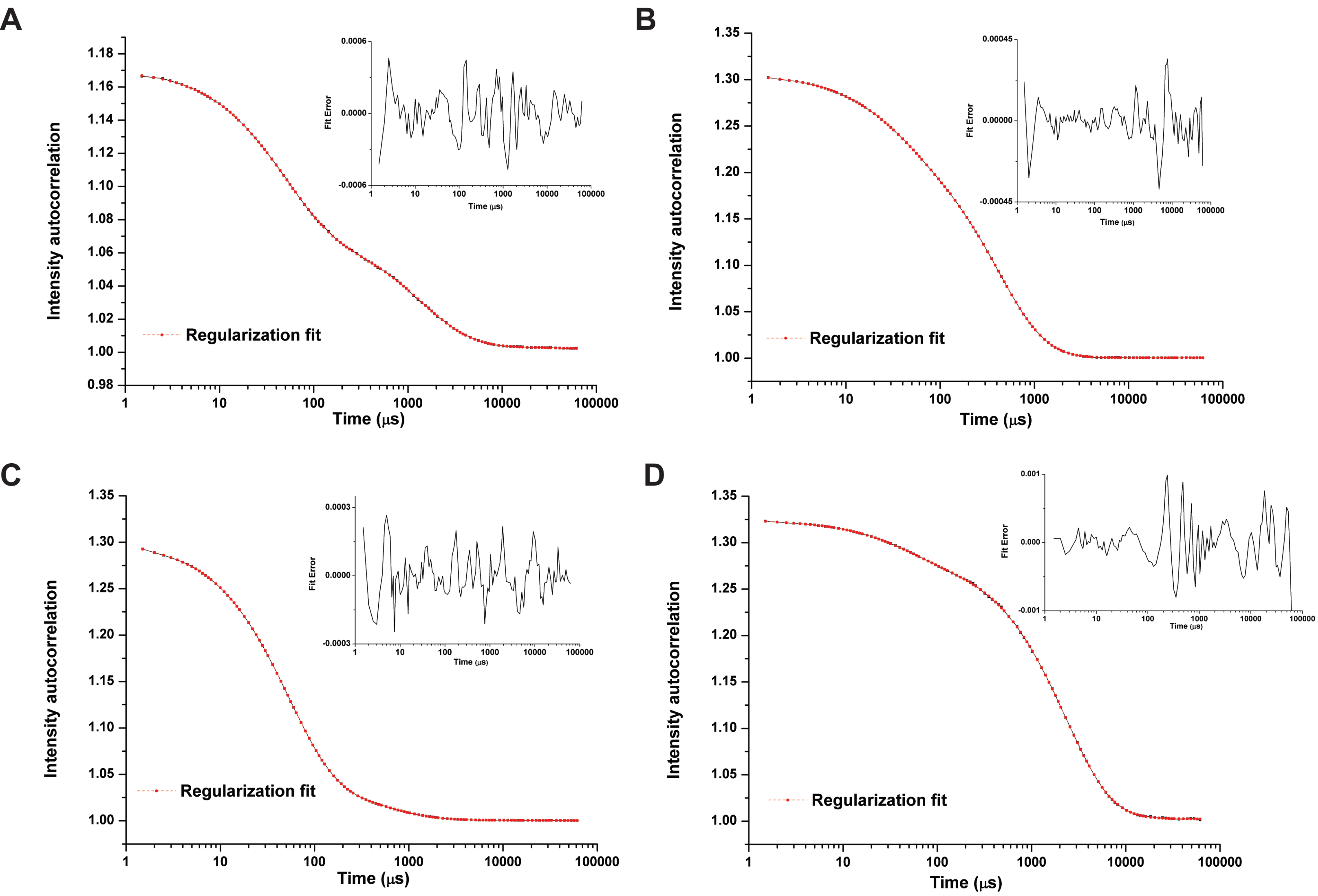
A
Intensity autocorrelation
Time (µs)
Regularization fit
Fit Error
Time (µs)
B
Intensity autocorrelation
Time (µs)
Regularization fit
Fit Error
Time (µs)
C
Intensity autocorrelation
Time (µs)
Regularization fit
Fit Error
Time (µs)
D
Intensity autocorrelation
Time (µs)
Regularization fit
Fit Error
Time (µs)

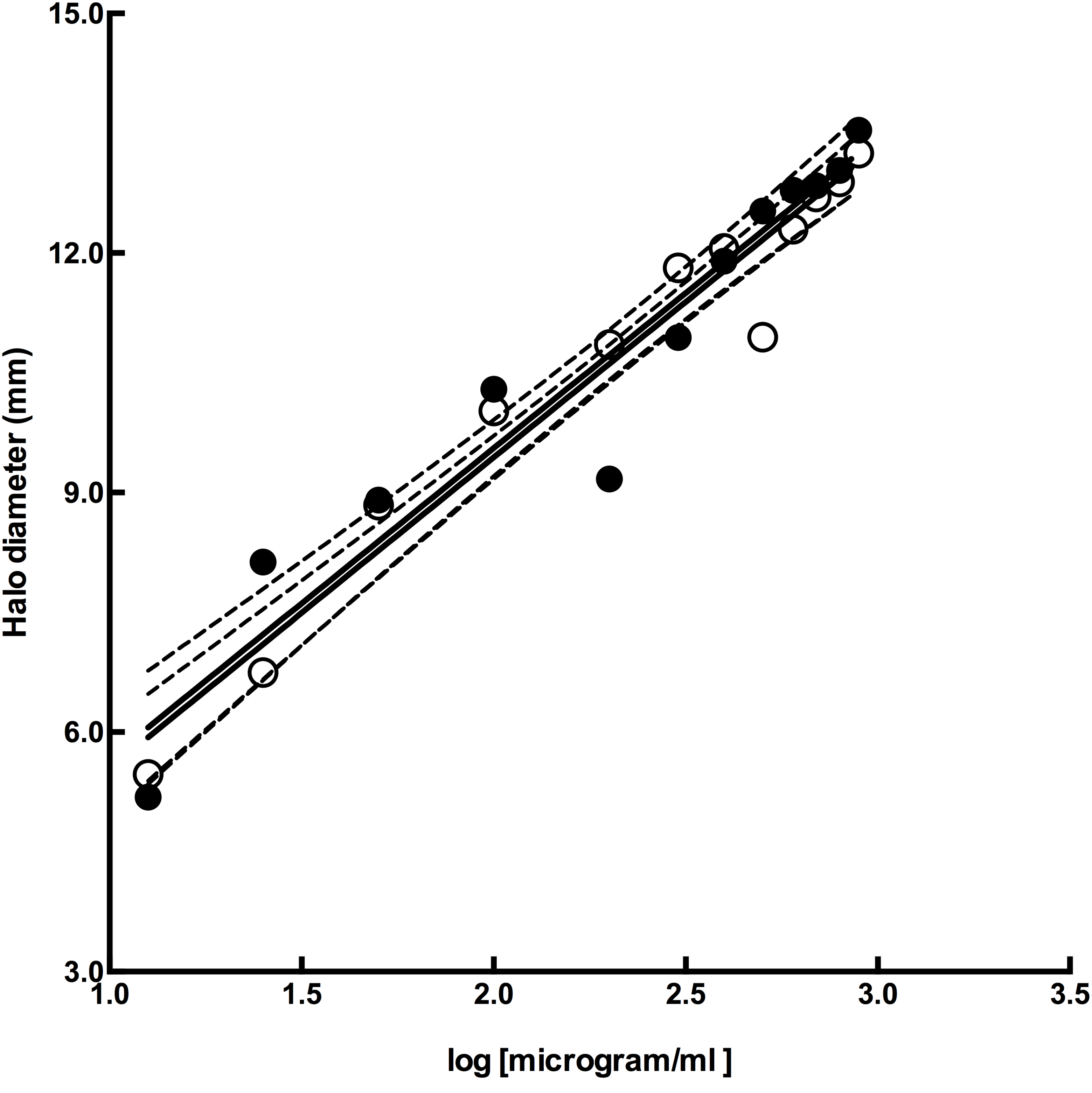
Halo diameter (mm)
15.0
12.0
9.0
6.0
3.0
1.0
1.5
2.0
2.5
3.0
3.5
log [microgram/ml]

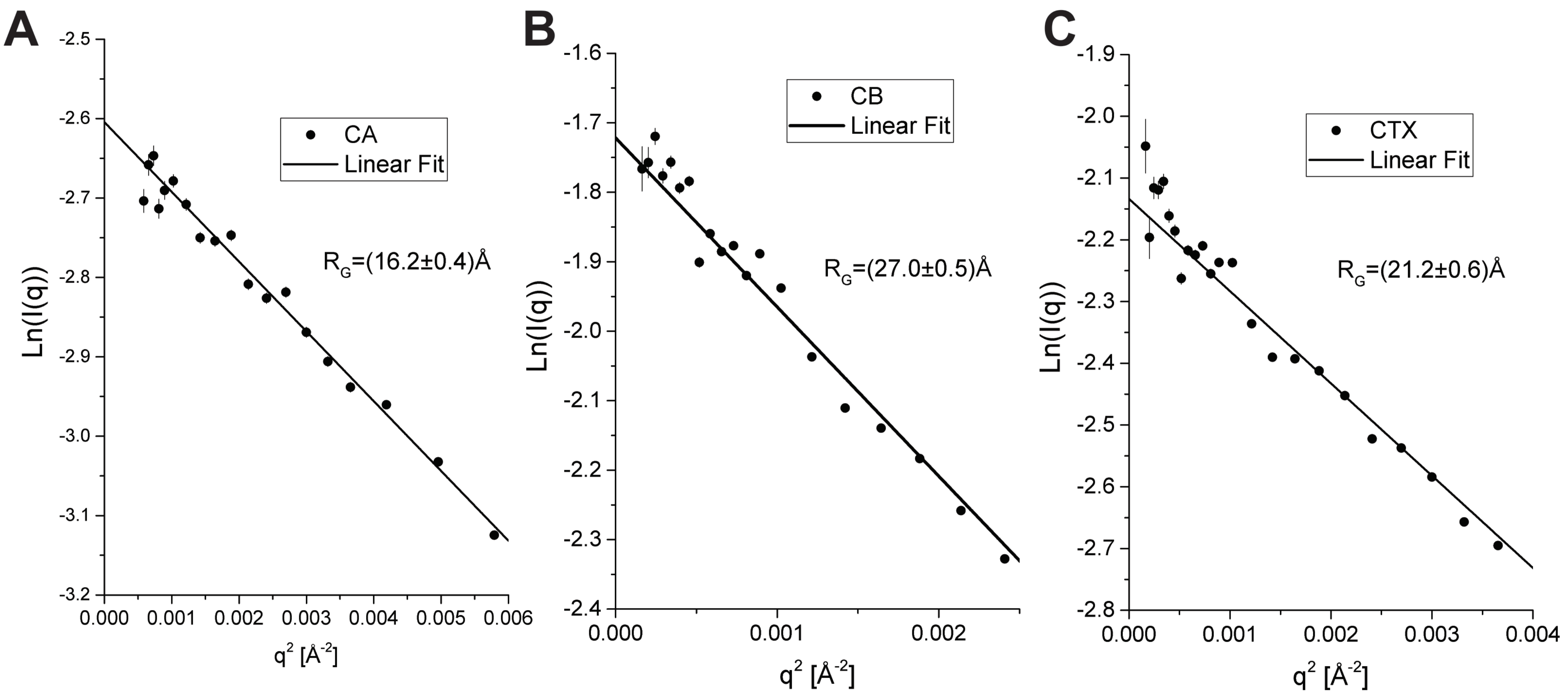
A
B
C
CA
CB
CTX
Linear Fit
Linear Fit
Linear Fit
R$_G$=(16.2±0.4)Å
R$_G$=(27.0±0.5)Å
R$_G$=(21.2±0.6)Å
Ln(I(q))
Ln(I(q))
Ln(I(q))
q^2 [Å^{-2}]
q^2 [Å^{-2}]
q^2 [Å^{-2}]
-2.5
-2.6
-2.7
-2.8
-2.9
-3.0
-3.1
-3.2
0.000
0.001
0.002
0.003
0.004
0.005
0.006
-1.6
-1.7
-1.8
-1.9
-2.0
-2.1
-2.2
-2.3
-2.4
0.000
0.001
0.002
-1.9
-2.0
-2.1
-2.2
-2.3
-2.4
-2.5
-2.6
-2.7
-2.8
0.000
0.001
0.002
0.003
0.004

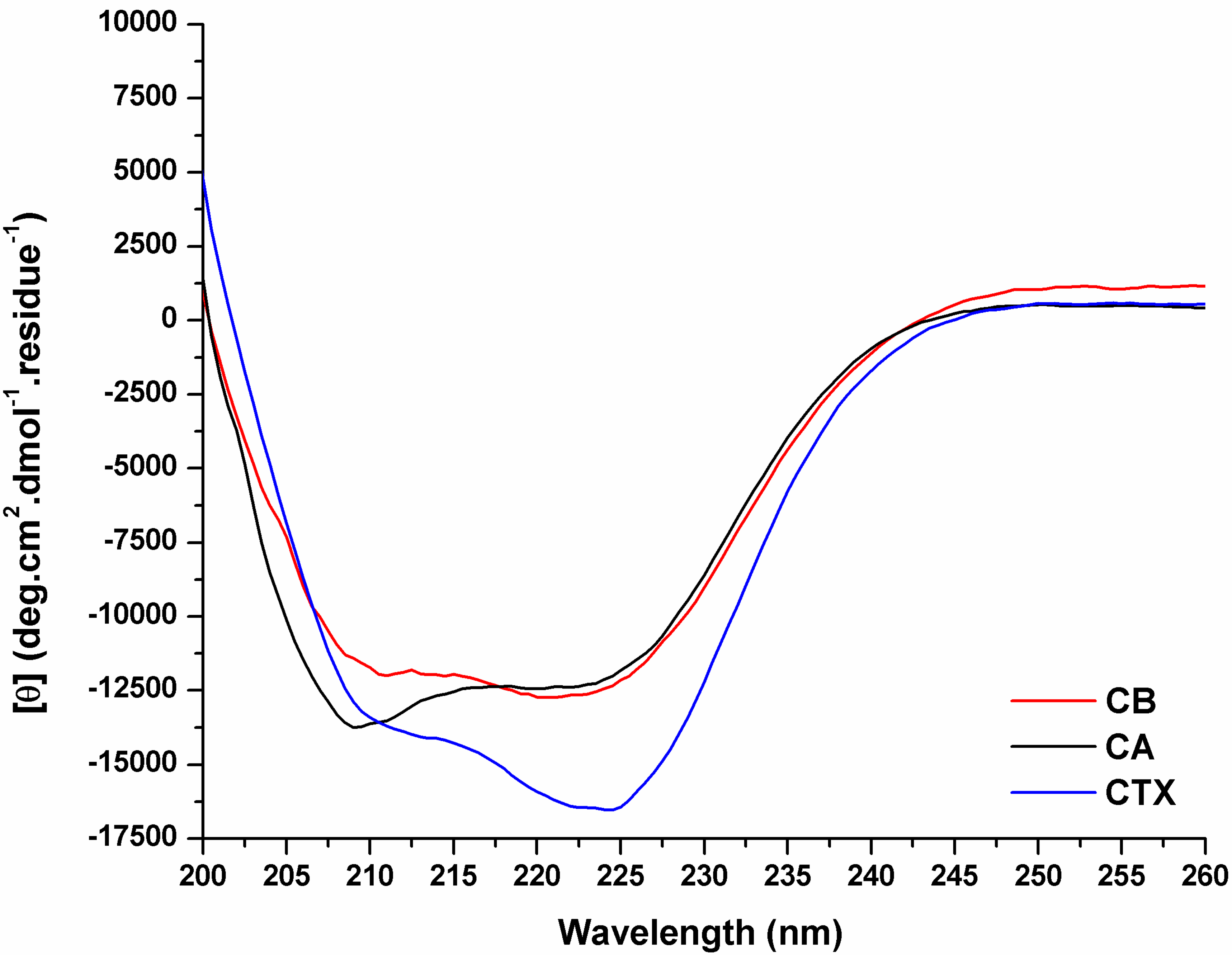

10000
7500
5000
2500
0
-2500
-5000
-7500
-10000
-12500
-15000
-17500
[θ] (deg.cm^2.dmol^{-1}.residue^{-1})
200
205
210
215
220
225
230
235
240
245
250
255
260
Wavelength (nm)
CB
CA
CTX

Supplementary Table 1

λ_{em}	**CA**	**CB**	**CTX**
335	2.70	3.33	1.73
340	2.74	3.35	1.85
345	2.75	3.45	1.90
350	2.77	3.46	1.99
355	2.82	3.54	2.03
360	2.85	3.56	2.13
365	2.88	3.58	2.13
